# CAPTURING IMPACT ON STUDENTS PARTICIPATING IN AGING IN PLACE: A PROGRAM TO ENHANCE GERIATRIC EDUCATION

**DOI:** 10.1093/geroni/igz038.2798

**Published:** 2019-11-08

**Authors:** Stephen T Smilowitz, Elizabeth O’Toole, Diana L Morris, Todd Fennimore, Cynthia Booth-Lord, Elizabeth Smilovich-Fine, David M Rosenberg, Patricia A Thomas

**Affiliations:** 1 Case Western Reserve University School of Medicine, Cleveland, Ohio, United States; 2 MetroHealth/Case Western Reserve University School of Medicine, Cleveland, United States; 3 Case Western Reserve University Frances Payne Bolton School of Nursing, Cleveland, United States; 4 Case Western Reserve University, Cleveland, Ohio, United States

## Abstract

Curricula to enhance healthcare students’ geriatric training has been lacking. Therefore, we developed AIP, an interprofessional (IP) community-based curriculum, in which IP student teams visit community-dwelling older adults. Using established instruments did not capture personal and professional changes experienced by students. Thus, an additional method using qualitative analyses of students’ six post-visit reflections over 15 weeks was employed to evaluate students ‘experiential learning. A grounded theory approach was used to describe students’ growth in geriatric proficiencies related to participation in the January-April 2017 AIP program . By program completion, 21 students had submitted 111 reflective essays. An interdisciplinary panel reviewed a sample of reflections and developed an initial coding system, which was then systematically applied to the whole via QSR-NVivo. Seventy-three distinct codes across 111 student essays generated 2515 occurrences. Prevalent themes, revealed by frequency analysis, and themes with remarkable trendlines yielded fifteen central themes. Students became attuned to their client’s life-world (n=185) as demonstrated by four central themes: 1) isolation, loneliness, and depression (n=44); 2) risks of fall (n=19); 3) loss of function/control (n=98); and, 4) importance of socializing in care (n=24). This attunement informed interactional intentionality (n=284), which shaped interactions with their client (n=207). From these authentic encounters, students described learning about 1) myself; 2) current and future practice; 3) team dynamics; and 4) my client as an older person. Systematic analysis of student reflections revealed student growth attributable to AIP. This evaluation approach should be further assessed in geriatric curricula.

